# The presence of bacteria varies between colorectal adenocarcinomas, precursor lesions and non-malignant tissue

**DOI:** 10.1186/s12885-019-5571-y

**Published:** 2019-04-29

**Authors:** Caspar Bundgaard-Nielsen, Ulrik T. Baandrup, Lars P. Nielsen, Suzette Sørensen

**Affiliations:** 1 Centre for Clinical Research, North Denmark Regional Hospital, Hjørring, Denmark; 2 Department of Clinical Medicine, Aalborg University, Aalborg, Denmark; 3 Department of Pathology, North Denmark Regional Hospital, Hjørring, Denmark; 4 Biobank and Biomarkers, Statens Serum Institut, Copenhagen, Denmark

**Keywords:** Colorectal cancer, Cancer microbiota, Colorectal adenomas, Fusobacterium, Bacteroides, Prevotella, Streptococcus, Acinetobacter

## Abstract

**Background:**

A causal association has been suggested between certain bacteria and colorectal cancer (CRC). Only a few studies have, however, investigated the presence of these bacteria directly in colon tissue with conflicting results. It is thus uncertain which role they may have in prognosis and carcinogenesis of CRC.

**Methods:**

Formalin-fixed and paraffin-embedded (FFPE) colorectal tissue samples from patients diagnosed with colorectal cancer (CRC)(tumor and paired normal tissue, *n* = 99), adenomas (*n* = 96), or diverticular disease (*n* = 104) were tested for the presence and bacterial load of *Streptococcus gallolyticus (S. gallolyticus)*, *Fusobacterium nucleatum (F. nucleatum)*, *and Bacteroides fragilis (B. fragilis)* using quantitative PCR. A subsequent broader search was conducted on a subset of samples using 16S ribosomal RNA gene sequencing. Finally, to evaluate the prognostic value, the bacterial status was compared to patient outcome.

**Results:**

*S. gallolyticus* was not detected by qPCR in any of the investigated tissue samples and *F. nucleatum* and *B. fragilis* were found to be equally distributed in tumors, paired normal tissue, and diverticula, but significantly less present in adenomas compared to both tumors and diverticula. Neither, *F. nucleatum* nor *B. fragilis* status affected the five-year prognosis of the patients. The 16S rRNA gene sequencing data revealed that tumors were associated with the *Prevotella* genus while conversely adenomas and diverticula were associated with *Acinetobacter* genus.

**Conclusion:**

These findings do not support a role of *F. nucleatum* or *B. fragilis* during colorectal beginning, while *S. gallolyticus* was not implicated in the colorectal tissue of a Danish population. A potential role of the bacterial genera *Prevotella* and *Acinetobacter* was indicated, and requires further investigations.

**Electronic supplementary material:**

The online version of this article (10.1186/s12885-019-5571-y) contains supplementary material, which is available to authorized users.

## Background

Colorectal cancer (CRC) is one of the most common cancers, with approximately 1.4 million cases diagnosed and 700.000 deaths reported annually worldwide [1]. CRC originates from mutations causing abnormal proliferation in the colorectal epithelium and subsequent formation of an adenomatous growth (adenoma) [2]. Through accumulation of mutations, such adenomas may lead to CRC [3, 4]. Several risk factors are associated with development of CRC, including diet, smoking and high alcohol consumption [5–9]. Early detection allows efficient treatment of CRC, but only 40% of cases are detected in early stage [10]. To improve diagnostics, screening systems for CRC have been implemented in many countries, where stool samples are analyzed for the presence of occult blood [11]. This, unfortunately, leads to a high number of false positive cases resulting in negative psychosocial consequences, increased costs, discomfort and complications related to follow-up diagnostic investigations [12]. Therefore, more research is needed, in order to find sensitive biomarkers for early non-invasive CRC detection.

A possible role for oncogenic bacteria in CRC was first noted in 1951 [13] and again in 1974 when it was shown that 64% of patients suffering from *Streptococcus bovis*-related endocarditis, also had colonic adenomas or CRC [14]. It was later revealed that the *Streptococcus bovis* subtype, *Streptococcus gallolyticus subsp. gallolyticus* (*S. gallolyticus*) had a uniquely strong correlation with CRC. Despite clinical associations [14–18], investigations of the prevalence of *S. gallolyticus* infection directly in CRC tumors have shown conflicting results [19, 20]. Recent studies have demonstrated enrichment with the bacteria *Fusobacterium nucleatum* (*F. nucleatum*) [20–26] and *Bacteroides fragilis* (*B. fragilis*) [27–30] in tumor tissue and fecal material of CRC patients, while a subsequent investigation indicated that high-level colonization with *F. nucleatum* or *B. fragilis* were indicators of poor prognosis in CRC patients [31]. To understand the role of bacteria in colorectal carcinogenesis, we investigated the bacterial involvement in the healthy tissue-adenoma-carcinoma sequence of CRC development. Previous studies investigating precancerous adenomas, have found diverging bacterial compositions. Enrichment of *F. nucleatum* has been documented in both fecal samples from patients with adenomas [32–34] and directly in biopsies from the adenomas [32, 35, 36]. Conversely, Pagnini et al. [37] found a marked reduction of mucosal adherent bacteria in adenomas, while Shen et al. [38] did not detect *F. nucleatum* in adenomas but only in biopsies from healthy volunteers. A recent study by Rezasoltani et al. [34] demonstrated enrichment of *F. nucleatum, B. fragilis* and *S. bovis* in tubular, villous and tubulovillous adenomas but not in hyperplastic or serrated polyps, while in contrast, Yu et al. [39] found serrated polyps to be more frequently enriched with *F. nucleatum* compared to tubular adenomas. While a gradual increase in enrichment of *F. nucleatum* from healthy colorectal tissue to adenomas and finally to CRC has been demonstrated [32, 33, 36, 40, 41], less is known for *B. fragilis* [42] or *S. gallolyticus*.

The majority of studies investigating bacterial involvement in the adenoma-carcinoma sequence were based on fecal samples [32, 33, 40, 41]. Fecal samples are plentiful and are thus often used as a non-invasive method for investigating gut microbiota. Some variations can however, be observed between fecal microbiota and the microbiota of the mucosal lesion [43]. As a result, more information is needed concerning enrichment of *S. gallolyticus*, *F. nucleatum* and *B. fragilis* in mucosal samples during the colorectal adenoma-carcinoma sequence. Formalin-fixed and paraffin embedded (FFPE) tissue blocks may serve as an abundant source of tissue, enabling studies on bacterial involvement directly in the colorectal tissue. In this study, we compared bacterial colonization of archival colorectal tissue from non-cancerous tissue, adenomas and tumors. Furthermore, we investigated the effects of bacterial status on patient outcome.

## Methods

### Sample selection

Using the National Pathology Data Bank, we identified all patients diagnosed with colorectal adenocarcinoma, colorectal adenomas, and diverticular disease at the Department of Pathology, North Denmark Regional Hospital in the period 2002–2010. Following surgical removal, tissue samples were stored as FFPE tissue using standard procedures for the Department of Pathology. Number of samples included was based on sample size calculations for two proportions [44], using a power of 80%, level of confidence of 95% and published prevalences of *S. gallolyticus* [19], *F. nucleatum* [25], and *B. fragilis* [20] in tumor tissue compared to non-neoplastic surrounding tissue. Patients diagnosed with more than one of the investigated lesions were excluded. Samples containing too low DNA concentrations or non-amplifiable DNA were excluded. We collected FFPE tissue from 99 patients diagnosed with colorectal adenocarcinoma (tumors and non-neoplastic paired normal tissue), 96 patients diagnosed with colorectal adenomas, and 104 patients diagnosed with diverticular disease of the colon. An overview of samples can be seen in Additional file 1. Paired normal tissue was only routinely collected from tumors, and thus no paired normal samples were available from diverticula or adenomas. All samples were stored using standard procedures at the Department of Pathology.

### Sample preparation and DNA extraction

FFPE samples were collected, with each sample, including tumors and paired normal tissue, occupying separate paraffin blocks. Consecutive tissue sections were prepared from all tissue blocks in the following order: 1 × 4 μm sections for HE (Hematoxylin and Eosin) staining, 4 × 10 μm for DNA purification, and finally 1 × 4 μm sections for comparative HE staining to ensure uniformity and for evaluation by a trained pathologist. This microscope based evaluation revealed neoplastic cells in 23 samples of paired normal tissue and these were therefore excluded, resulting in a total of 99 tumor tissue but only 76 paired normal tissue samples being included. To minimize the risk of cross-contaminations between samples, section knives were changed after each tissue block, and the microtome surface wiped clean with alcohol and RNase Away (Molecular Bioproducts). To monitor potential carry-over of bacterial DNA between samples, an empty paraffin block was included for every 11th patient tissue sample. This paraffin block was freshly prepared but otherwise handled similar to blocks containing tissue.

DNA was isolated from FFPE tissue sections using the AllPrep® DNA/RNA FFPE kit (Qiagen), according to manufacturer’s instruction.

### Primer design and qPCR amplification and quantification

Quantitative real time polymerase chain reaction (qPCR) was used to investigate presence and quantity of bacterial species previously associated with CRC in the different histological tissue types. Primers targeting *S. gallolyticus* species, *S. gallolyticus* subspecies *gallolyticus*, *F. nucleatum,* and *B. fragilis* were designed in-house using Primer3 software, and tested for specificity using primer-BLAST (NCBI) [45]. The qPCR sought to determine how the relative abundance of *S. gallolyticus*, *F. nucleatum* and *B. fragilis* differed between different histological tissue types, and thus a reference gene was designed targeting the human β-actin gene. Since DNA extracted from FFPE tissue tends to be fragmented [46], we aimed for amplicon sizes shorter than 200 bp. The sequences, targets, and parameters of the individual primers are summarized in Table 1.

**Table 1:** Primers used for 16S rRNA gene sequencing and qPCR analysis

Target	Target gene (NCBI Accession number)	Primer sequence	Tm (°C)	Product (bp)
Bacteria and Archaea (sequencing)	V4 variable region of the 16S rRNA (515F and 806R [51])	F: 5′-GTGCCAGCMGCCGCGGTAA-3′ R: 5′-GGACTACHVGGGTWTCTAAT-3´	65.4 49.0	~ 250–390 bp^a^
*S. gallolyticus* spp.	SodA (AP012053) (HE613569) (AP012054)	F: 5´-GCTTGGCTTGTGGTGAATGA-3′ R: 5′-GCGAACGTTGCGATACTTGA-3´	59.0 59.3	144
*S. gallolyticus* subsp. *gallolyticus*	SodA (AP012053)	F: 5´- AAGCTGCGACAACTCGCTTT − 3′ R: 5′- AAGCGTGTTCCCAAACGTCA − 3´	61.1 60.8	150
*F. nucleatum*	16 s ribosomal RNA (CP012717)	F: 5´–CCCAAGCAAACGCGATAAGT–3′ R: 5´–GCGTTGCGTCGAATTAAACC–3´	59.2 58.9	117
*B. fragilis*	16 s ribosomal RNA (M11656)	F: 5′- AGTAGAGGTGGGCGGAATTC − 3′ R: 5′- GTGTCAGTTGCAGTCCAGTG − 3´	59.2 59.1	97
β-actin	β-actin (NG_007992)	F: 5´-ACTCGTCATACTCCTGCTTGC-3′ R: 5′-CCTCCTCAGATCATTGCTCCTC-3´	60.1 60.0	118

^a^Amplicon length varies depending on target bacteria [51]

Bacterial DNA was purchased from DSMZ (Leibniz Institute DSMZ-German Collection of Microorganisms and Cell Cultures), including DNA from *S. gallolyticus* subspecies *gallolyticus (*DSM 16831*)*, *S. gallolyticus* subsp. *macedonicus (*DSM 15879*)*, *S. gallolyticus* subspecies *pasteurianus (*DSM 15351*), F. nucleatum (*DSM 15643*),* and *B. fragilis (*DSM 2151*)*. The bacterial DNA was used for determining limit of detection (LOD) of the individual primers using a dilution series. This was found to be approximately 109 DNA copies for *S. gallolyticus spp.*, 10 DNA copies for *S. gallolyticus* subsp. *gallolyticus*, 12 DNA copies for *F. nucleatum*, and 10 DNA copies for *B. fragilis*. The bacterial DNA was further used as positive control for qPCR analyses by spiking bacterial DNA into human DNA samples extracted from FFPE colorectal tumors to mimic the sample types used in this study. The ratio of bacterial DNA to total human DNA was 1:40.

qPCR was performed using the Brilliant III Ultra Fast SYBR® Green QPCR Master Mix (Agilent Technologies) according to manufacturer’s recommendations, and analyzed on the Mx3005P qPCR System (Agilent Technologies). All experiments were performed in triplicates using 40 ng of input DNA with the following cycling conditions: 95 °C for 10 min, 40 cycles of 95 °C for 1 min, 55 °C for 30 s and 72 °C for 30 s. In a few cases, several products were apparent on the melting curve analysis, and the PCR was then repeated using a more stringent annealing temperature of 59 °C.

For relative quantification of bacterial DNA in samples the ΔΔCt method [47] was applied, utilizing the primers summarized in Table 1, with β-actin serving as reference gene.

### Five year follow-up

The patient’s histological history was followed over a 5 year period using the National Pathology Data Bank. Time of death or occurrence of new cases of adenomas or cancer in the colorectum were noted for each patient. Survival and disease-free survival were analyzed using the Kaplan-Meier method based on detection of bacteria. Social security numbers were not available for two patients, and their clinical data were therefore not recorded.

### 16S rRNA gene sequencing

To detect other potential bacterial biomarkers, the composition of bacterial genera were analyzed using 16S ribosomal RNA (rRNA) gene sequencing in a subset of the FFPE samples already investigated in this study. A total of 40 tissue samples were chosen using the Research Randomizer software [48] to randomly select 10 samples belonging to each separate histological tissue group (Additional file 2). Bacterial 16S rRNA amplicon sequencing targeting the V4 variable region, was performed by DNAsense (Denmark), and followed a modified version of an Illumina protocol [49]. Briefly, an initial PCR and clean-up was performed as described by Albertsen et al. 2015 [50] using primers targeting the V4 hypervariable region (Table 1) [51], and 35 cycles of amplification. Next, indexing primers were attached to all sequences using a second PCR, followed by clean-up [49]. Finally, all samples were pooled and sequenced using a MiSeq (Illumina, USA) as previously described [52]. 20% PhiX control library (Illumina) was added to estimate error rate during sequencing, a negative control (nuclease-free water) was added to eliminate background while a positive control (complex sample obtained from an anaerobic digester system) were used to monitor sequencing efficiency and batch effects.

### Bioinformatics

Quality of reads were analyzed using FastQC (Babraham Bioinformatics, UK). Forward reads were trimmed using Trimmomatic v0.32 [53] to remove poor reads and reads shorter than 250 bp using the settings SLIDINGWINDOW:5:3 and MINLEN:250. The reads were next dereplicated and processed using the UPARSE workflow [54]. The initial 250 bp of all sequencing reads were clustered using the Usearch v. 7.0.1090 -cluster_otus command with default settings. Operational taxonomic units (OTUs) were formed based on 97% identity and chimeras removed using the Usearch v. 7.0.1090 –usearch_global command with –id 0.97. Finally, taxonomy was assigned using the RDP classifier [55] as implemented in the parallel_assign_taxonomy_RDP.py script in QIIME [56] using the MiDAS database v. 1.20 [57].

### Statistics

Data analysis was performed using R version 3.5.2 [58] through the Rstudio IDE (http://www.rstudio.com/), and Microsoft Office Excel 2013. For continuous data, distributions were tested using Shapiro-Wilk test. 16S rRNA gene sequencing data was analyzed using the ampvis2 package v.2.3.11 [59] for Rstudio. α diversity was determined using OTU richness and Shannon diversity index as implemented in the amp_alphadiv command of the ampvis2 packet in R. β diversity was visualized using heat maps depicting the 20 most commonly found OTUs and explored using Principal component analysis (PCA) and redundancy analysis (RDA) clustering of Hellinger Distance transformed OTU abundances. Bacterial genera with statistical significant different distributions amongst differing tissue types, were identified using the DESeq2 package in Rstudio [60] to generate multiple hypothesis corrected *p*-values using the Benjamini-Hochberg procedure [61]. For a bacterial genus to be considered for further analysis, it needed to be significantly different between tissue groups, and the difference was required to be universal for the majority of samples in the tissue group. That is, for a bacteria to be considered associated with tumor tissue, it should constitute a statistically significant higher proportion of bacteria in the majority of tumor samples.

Categorical data, like presence or absence of bacteria, were analyzed using χ^2^ test. For continuous data like OTU richness and Shannon diversity index, distribution was tested using Shapiro-Wilks test while variance was tested using Bartlett’s test. Normal distributed data with equal variance were compared using ANOVA followed by Tukeys post-hoc test while non-parametric data were tested using Kruskal-Wallis test followed by Dunn’s post-hoc test. Finally, 5-year follow-up data were analyzed using the Kaplan-Meier method, and a log-rank test were used to compare outcome between patients positive and negative for bacterial infection.

A *p* value of < 0.05 was considered significant for all statistical tests, with the exception of multiple hypothesis corrected *p* values where a limit of < 0.01 was utilized.

## Results

### Demographic and histopathological description of patient samples

In this study, colon samples from four different histological tissue groups were analyzed. The demographic and histopathological characterization of these groups are presented in Tables 2 and 3 respectively. CRC patients (71 ± 10.1 years) were significantly older (*p* < 0.05) than adenoma (66 ± 11.7 years) and diverticulum patients (63 ± 14.0 years). While the location of tumor samples was more widely distributed, the majority of adenoma and especially diverticulum samples were localized in the left colon, constituting 48.5% of CRC tumor cases, 76.0% of adenoma cases and 89.4% of diverticulum cases. Information concerning age group, gender, histologic tissue group and follow up data can be found in Additional file 1.

**Table 2:** Demographic characterization of the individual patient groups

Feature	CRC (Tumors)		CRC (paired normal tissue)^a^		Adenomas		Diverticula	
Number of samples	99		76^b^		96		104	
Age (median)	71^c^		70.5^c^		66		63	
Standard deviation	± 10.1		± 9.9		± 11.7		± 14.0	
Age groups (%)	No.	(%)	No.	(%)	No.	(%)	No.	(%)
< 40	0	(0.0)	0	(0.0)	1	(1.0)	3	(2.9)
40–49	2	(2.0)	1	(1.3)	6	(6.3)	16	(15.4)
50–59	8	(8.1)	5	(6.6)	18	(18.8)	24	(23.1)
60–69	35	(35.4)	28	(36.8)	30	(31.3)	25	(24.0)
70–79	28	(28.3)	22	(28.9)	25	(26.0)	17	(16.3)
80–89	22	(22.2)	16	(21.1)	15	(15.6)	19	(18.3)
> 90	4	(4.0)	4	(5.3)	1	(1.0)	0	(0.0)
Gender (%)	No.	(%)	No.	(%)	No.	(%)	No.	(%)
Male	44	(44.4)	41	(53.9)	47	(48.0)	55	(52.9)
Female	55	(55.6)	35	(46.1)	51	(52.0)	49	(47.1)

^a^Healthy colon mucosa surrounding the tumor tissue. ^b^ 23 samples were removed following examination of the HE stained sections by a trained pathologist, due to presence of cancer cells. ^c^patients donating tumors and paired normal tissue are significantly older than those with adenomas or diverticula

**Table 3:** Histopathological description of the four tissue groups

Tissue group	Location^a^			Disease State		
CRC (tumor) (n = 99)	Location	n	(%)	Tumor stage^b^	n	(%)
	Right colon	49	(49.5%)	* I*	7	(7.1%)
	Left colon	48	(48.5%)	* II*	41	(41.4%)
	Rectum	2	(2.0%)	* III*	43	(43.4%)
	* Not reported*	0	(0.0%)	* IV*	8	(8.1%)
CRC (paired normal tissue) (*n* = 76)	Location	n	(%)	Tumor stage^b^	n	(%)
	Right colon	41	(53.9%)	* I*	7	(9.2%)
	Left colon	34	(44.7%)	* II*	29	(38.2%)
	Rectum	1	(1.3%)	* III*	32	(42.1%)
	* Not reported*	0	(0.0%)	* IV*	8	(10.5%)
Adenomas (n = 96)	Location	n	(%)	Subtype	n	(%)
	Right colon	15	(15.6%)	* Villous*	1	(1.0%)
	Left colon	73	(76.0%)	* Tubulovillous*	6	(6.3%)
	Rectum	3	(3.1%)	* Tubular*	89	(92.7%)
	*Not reported*	5	(5.2%)	Dysplasia	n	(%)
				* Mild*	11	(11.5%)
				* Moderate*	55	(57.3%)
				* Severe*	30	(31.3%)
Diverticula (n = 104)	Location	n	(%)	Inflammation	n	(%)
	Right colon	4	(3.8%)	* Diverticulosis*	23	(22.1%)
	Left colon	93	(89.4%)	* Diverticulitis*	81	(77.9%)
	Rectum	0	(0.0%)			
	* Not reported*	7	(6.7%)			

^a^Location of tissue samples in the colorectal tract, based on the proposed distinction described by Bufill et al. [73]. ^b^ Tumor classification based on the recommendations of International Union Against Cancer [74]

### *S. gallolyticus* was not detected in any of the investigated tissue groups

To establish whether CRC was associated with *S. gallolyticus*, we utilized qPCR to compare the prevalence and quantity of the bacteria in colorectal tumors, paired normal tissue, adenomas and diverticula. Surprisingly, *S. gallolyticus* was below the LOD for both primers targeting all *S. gallolyticus* spp. as well as the more sensitive primers specifically targeting *S. gallolyticus* subsp. *gallolyticus*, in all tissue types (see Additional file 1).

### *F. nucleatum* and *B. fragilis* were enriched in tumors compared to adenomas, but not paired normal tissue or diverticula

**Fig. 1:**
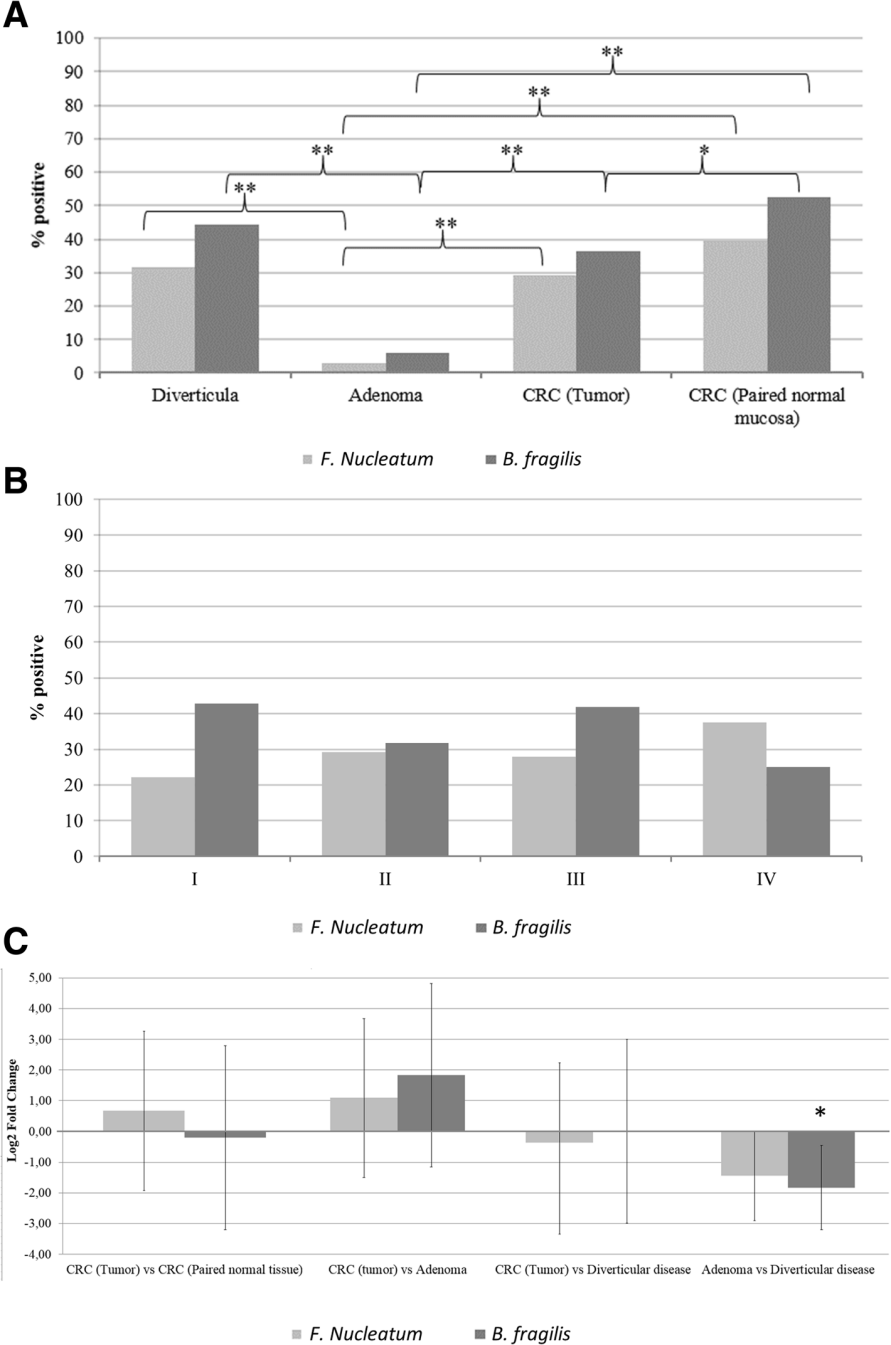
Presence and quantity of *F. nucleatum* and *B. fragilis* in colorectal tissue. qPCR determination of presence and quantity of bacterial DNA in 99 colorectal tumor tissue, 76 paired normal tissue, 96 adenomas and 104 diverticula. **a** Prevalence of *F. nucleatum* and *B. fragilis* in colorectal tissue. Positivity was determined as bacterial species with a DNA quantity above the LOD of the primers. **b** Prevalence of *F. nucleatum* and *B. fragilis* in different stages of CRC. No statistical significant differences were observed. **c** Difference in quantity of *F. nucleatum* and *B. fragilis* DNA in colorectal tumor tissue compared to paired normal tissue, adenomas and diverticula as well as in adenomas compared to diverticula. Brackets denote standard deviation. * *P* < 0.05, ** *P* < 0.001

To establish the degree of colonization with *F. nucleatum* and *B. fragilis* at different stages in the colorectal adenoma-carcinoma sequence in CRC, we utilized qPCR to compare the prevalence and quantity (Fig. 1) of the bacteria in colorectal diverticula, adenomas, tumors and paired normal tissue. For all empty paraffin blocks, the quantity of the tested bacteria were below the LOD of the primer, indicating that no cross-contamination occurred.

*F. nucleatum* could be detected in 29.3% of tumor samples and *B. fragilis* in 36.4% of cases (Fig. 1a). These distributions were comparable to those found in paired normal tissue and non-malignant diverticula, except for *B. fragilis* which was detected in a higher proportion of paired normal tissue samples (52.6%, *p* < 0.05). The presence of *F. nucleatum* and *B. fragilis* were furthermore comparable when stratifying tumor samples based on cancer stages (Fig. 1b). For tumors, paired normal tissue and diverticula, the bacterial loads of *F. nucleatum* and *B. fragilis* were comparable (Fig. 1c). Intriguingly, we detected *F. nucleatum* and *B. fragilis* significantly less common in adenoma tissue (3.0 and 5.9% respectively) compared to both tumor tissue (29.3 and 36.4%, *p* < 0.001) and diverticula (31.7 and 44.2%, p < 0.001)(Fig. 1a). In addition, the adenomas contained significantly less *B. fragilis* DNA compared to diverticula (p < 0.05)(Fig. 1c).

Overall, neither *F. nucleatum* nor *B. fragilis* were found to be specifically associated with tumors of CRC patients, but both bacteria were noted by their low presence in adenomas.

### *F. nucleatum* and *B. fragilis* status do not affect survival or disease-free survival of patients over a five year period

**Fig. 2:**
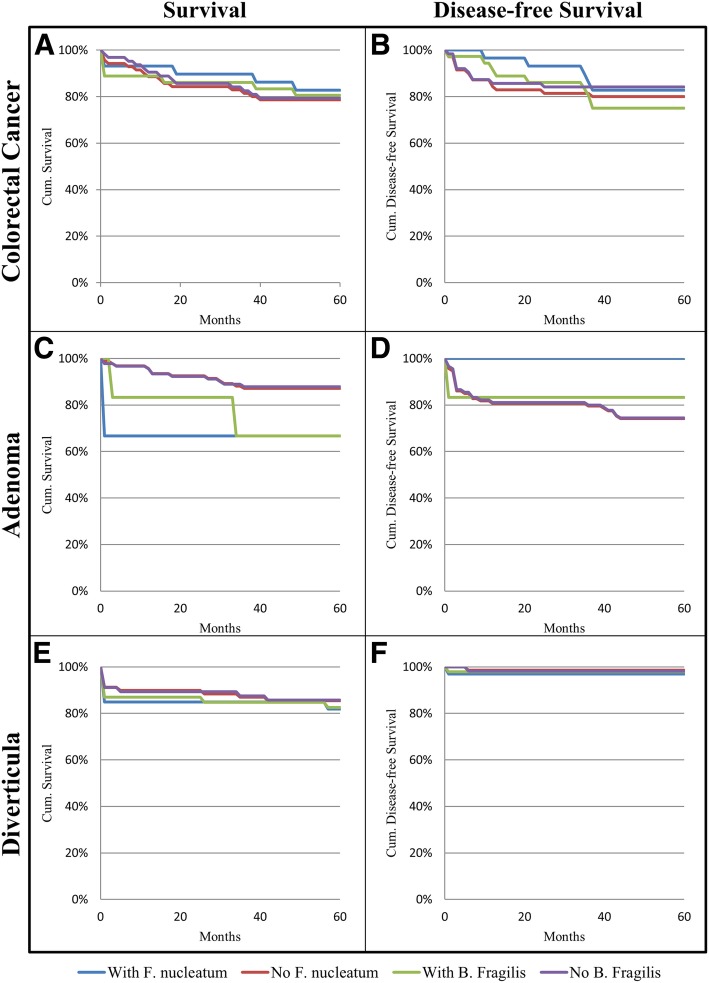
Five-year follow-up based on presence of *F. nucleatum* or *B. fragilis*. Survival (**a**, **c**, **e**) and disease-free survival (**b**, **d**, **f**) of patients presenting with CRC (**a** and **b**, *n* = 99), adenomas (**c** and **d**, *n* = 96) or diverticula (**e** and **f**, *n* = 104) depending on presence or absence of *F. nucleatum* or *B. fragilis*. Five-year follow-up data was not available for two study participants belonging to the diverticula group. These patients were excluded from the follow-up analysis

To assess the clinical significance of *F. nucleatum* and *B. fragilis*, information on disease progression and survival were collected for all patients for a 5 year period following initial diagnosis. A Kaplan-Meier analysis was performed to examine the relationship between bacterial status in the investigated patients, with survival and risk of developing new cases of CRC or adenomas (Fig. 2). Detection of *F. nucleatum* or *B. fragilis* did not result in significant (*p* > 0.05) changes in survival or disease-free survival rates of patients within a 5 year period.

### Bacterial composition of tumor tissue overlaps with that of paired normal tissue, but differs from adenoma and diverticula

To determine if CRC tissue from the four groups (CRC tumors, paired normal tissue, diverticula, and adenomas) differed in overall bacterial composition, we applied a more global approach using 16S rRNA gene sequencing. A subsection of 10 samples from each group were randomly selected and analyzed. Following quality filtering and chimera removal, 566,527 16S rRNA sequence reads (mean number per sample: 16,186.5 ± 4814 reads) were obtained. A total of 696 unique Operational Taxonomic Units (OTUs) were identified, with 97.99% being identified on the phylum taxonomic level and 63.51% on the genus level. One sample from the adenomas and four paired normal tissue yielded less reads than the negative controls (3045 and 3123 reads) and were thus excluded, resulting in a total of 35 samples (10 tumors, 6 paired normal tissue, 9 adenomas and 10 diverticula) being analyzed. A rarefication curve was produced, showing good sequencing coverage (data not shown).

**Fig. 3:**
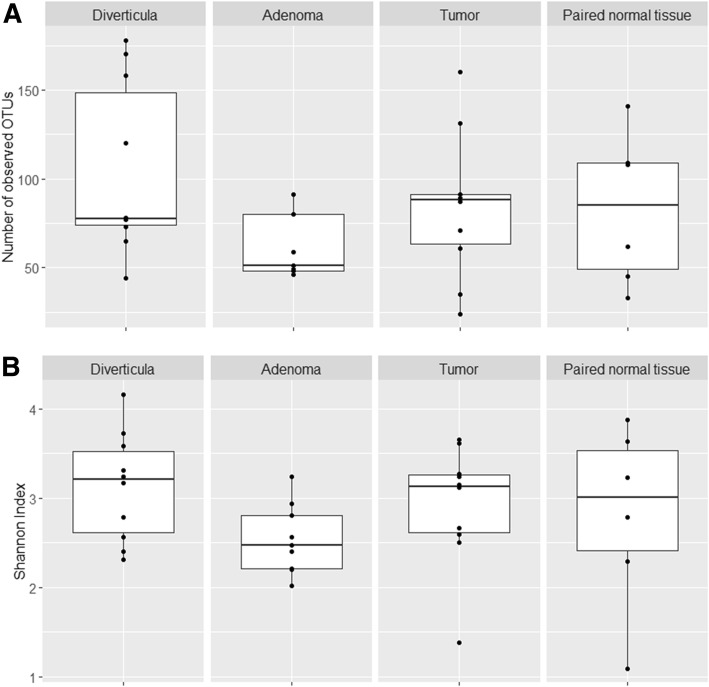
Gut microbiome richness and diversity between tissue types in a subsection of samples. **a** OTU richness and **b** Shannon diversity index was compared between a subsection of the tumors, paired normal tissue, adenomas and diverticula included in this study. A total of 35 tissue samples were investigated, with 10 tumors samples, 6 paired normal tissue, 9 adenomas and 10 diverticula

**Fig. 4:**
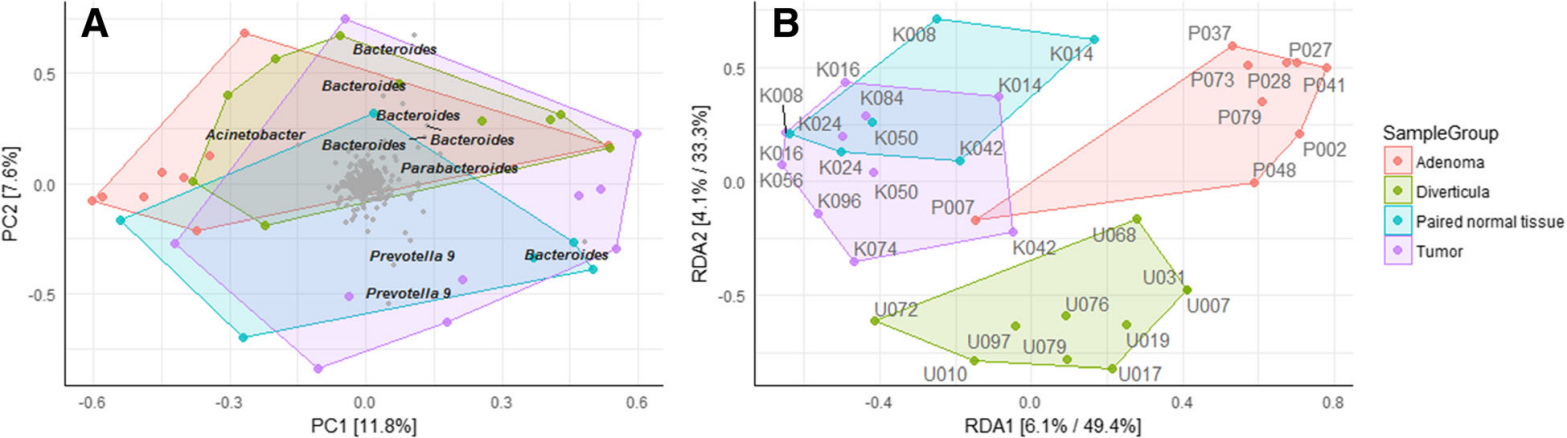
Variation in bacterial composition between individual samples and tissue types. β-diversity was investigated in 35 tissue samples using **a**) PCA and subsequent **b**) RDA plots with Hellinger Distance of OTU abundances. Colored boxes represent different tissue types

We first investigated bacterial richness and diversity of the four tissue types (Fig. 3). No significant differences were observed between either tissue types for either OTU richness (Fig. 3a) or Shannon diversity index (Fig. 3b), although diverticula had a slightly higher OTU richness compared to all other tissue types. β diversity was established to determine differences and similarities in bacterial composition between the tissue types. Interestingly, the differences between bacterial compositions were minor as indicated by clustering on the PCA plot (Fig. 4a). These minor changes were elucidated through a subsequent RDA plot that reveal limited tissue specific clustering (Fig. 4b). Tumor tissue clustered separately from diverticula and adenoma tissue, but was highly similar to paired normal tissue.

### Differences in proportion of *Prevotella* and *Acinetobacter* defines tissue from CRC samples versus adenoma and diverticula

**Fig. 5:**
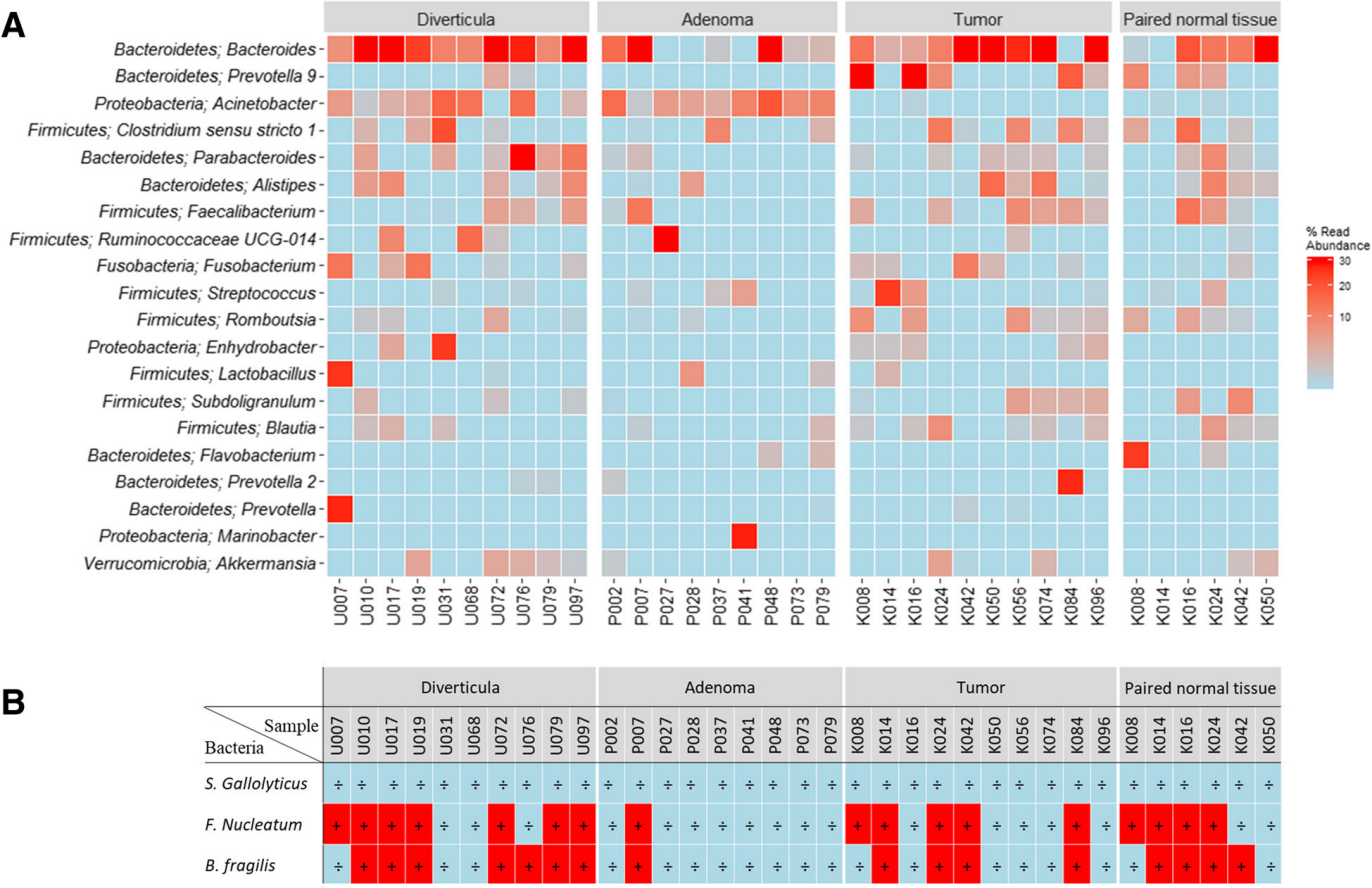
Bacterial composition in a subsection of tissue types. The bacterial composition of the 35 samples analyzed using 16S rRNA gene sequencing, was visualized. **a** Heatmap of the investigated samples. Colors represent bacterial composition, with stronger red indicating higher percentage of total read abundance, while light blue indicate absence of the bacteria. The 20 most common bacteria are depicted on the y axis, while the x-axis contains the 35 samples included in this analysis. **b** qPCR results for the investigated subsection of tissue samples. Samples starting with “U” indicated diverticula, “P” indicate adenomas while samples starting with “K” indicate samples originating from patients diagnosed with CRC (tumors or paired normal tissue)

We next sought to identify the bacteria that differed between the investigated subsection of colorectal tissue samples (Fig. 5a). Of especial note are the genera *Prevotella* and *Acinetobacter*. *Prevotella* is a dominant bacteria in several samples from tumors and paired normal tissue, but markedly absent from especially adenoma but also diverticula (*p* < 0.01). *Acinetobacter* are conversely not represented in tumor or paired normal tissue, but are dominant in the majority of samples originating from adenomas or diverticula (p < 0.01). Despite the *Streptoccocus* species *S. gallolyticus* being below the LOD for all samples investigated in this study, the genus *Streptococcus* was significantly more common in tumor tissue compared to paired normal tissue, diverticula and adenomas (p < 0.01). The genus *Fusobacterium* was significantly more common in tumor tissue compared to adenomas (p < 0.01), but not paired normal tissue nor diverticula (*p* > 0.05), while no differences were observed in composition of the *Bacteroides* genus between any tissue types investigated (p > 0.05). We observed that a high composition of the genera *Fusobacterium* and *Bacteroides* using sequencing (Fig. 5a) did not clearly correlate with detection of the bacterial species *F. nucleatum* or *B. fragilis* using qPCR (Fig. 5b).

## Discussion

In recent years, there has been a growing number of reports concerning a possible link between different bacterial species and the development of CRC. Several bacteria have been implicated, including *S. gallolyticus* [15, 17–19], *F. nucleatum* [20–22, 62] and *B. fragilis* [27–29, 63]. To investigate changes in the bacterial composition along the colorectal healthy tissue-adenoma-carcinoma sequence, we performed qPCR and 16S rRNA gene sequencing on FFPE tissue from colorectal diverticula, adenomas, tumors and paired normal tissue.

Adenomas harbored a distinct bacterial community compared to non-malignant controls, which has been supported by others [35, 37, 38]. While the genus *Acinetobacter* constitutes a large percentage of total bacteria in both diverticula and adenomas, the relative abundance of *Bacteroides,* as well as the percentage of samples positive for the species *F. nucleatum* and *B. fragilis,* were reduced in adenomas compared to both diverticula and paired normal tissue. The cause for this different microbial composition is unknown, but may result from increased local inflammation during adenoma formation, as previously indicated [37]. This increased inflammation may result in development of a microbial community with oncogenic potential [42, 64]. Notably, not all adenomas transition into CRC [65], and it will therefore be interesting, to establish whether there exists different subtypes of adenomas with various bacterial compositions and potential of carcinogenic progression. During the colorectal adenoma-tumor sequence, we observed a marked increase in the relative abundance of the bacterial genus *Prevotella* as well as the species *F. nucleatum* and *B. fragilis*, all of which have previously been shown to be associated with colorectal tumors [24, 32, 33, 36, 40–42, 66, 67]. These bacteria are known to promote a pro-inflammatory environment [27, 32, 63, 68, 69], and may thus drive the adenoma-tumor transition by inducing local chronic inflammation. Conversely, we observed that bacteria belonging to the genus *Acinetobacter* were absent from all samples originating from patients diagnosed with CRC (both tumors and paired normal tissue), while being highly abundant in both diverticula and adenomas. Similar observations have been made in rectal cancer [70], and further suggests that a distinct bacterial niche develops during the adenoma-tumor transition. In contrast to previous studies [41, 42], we did not observe a difference in the percentage of early and late stage CRC tumor samples positive for *F. nucleatum* or *B. fragilis*, indicating that these bacteria do not drive tumor progression. Finally, to elucidate the role of *F. nucleatum* and *B. fragilis* in initiation and progression of CRC, we investigated the 5 year risk of new cases of adenomas, CRC or death depending on bacterial status. In our study neither *F. nucleatum* nor *B. fragilis* affected the risk of death or the risk of developing new adenomas or CRC in either CRC, adenoma or diverticular disease patients. Overall our results suggest that the bacterial genus *Prevotella* and the species *F. nucleatum* and *B. fragilis* may play a role in the transition of adenomas to CRC, but not in initiation of adenomas nor in the progression from early to late stage colorectal tumors.

Two surprising observations were noted during this study. First, despite the noted association with CRC [14, 19, 34, 71], we did not detect *S. gallolyticus* in any of the investigated tissue samples. The conflicting results could potentially be explained through ethnic differences in susceptibility to *S. gallolyticus* colonization of colorectal mucosa or geographical differences in *S. gallolyticus* distribution. This is supported by similar findings by Viljoen et al. [20] in a South African CRC population. Secondly, while several studies [21, 22], including the current study, utilize paired normal tissue obtained from CRC patients as a matched “healthy” control, we observed that the bacterial composition of tumor tissue and paired normal tissue overlapped considerably. While more samples are needed to validate this observation, it does question the validity of using paired normal tissue as healthy controls when investigating bacteria of CRC.

This study has a number of limitations. First, all samples used were fixed with formalin. Since formalin is known to affect DNA quality [72], this may have limited our ability to detect bacteria. Since all tissue samples were handled similarly, we do not expect the formalin fixation to affect the observed differences in bacterial load and prevalence between diagnoses. A second limitation involves the previously reported difficulties in extracting DNA from gram-positive bacteria like *S. gallolyticus* [50]. The sequencing data revealed a high proportion of gram positive bacteria including other members of the *Streptococcus* genus. Thus, this limitation does not explain the lack of *S. gallolyticus* reported in this study. Finally, while the primers used in this study have low LODs compared to bacterial DNA, the LODs were established on purified DNA from bacteria, which would have a higher quality compared to FFPE bacterial DNA stored for up to 10 years. The true LOD of the primers in the examined tissue samples, could therefore be higher, as reported by Viljoen et al. [20]. This could prevent detection of low abundance bacteria, causing us to underestimate the bacterial colonization across all samples. This study had a specific focus on the bacterial species *F. nucleatum*, *B. fragilis* and *S. gallolyticus*. However, other studies have revealed other bacteria with an unique correlation with CRC, including *Escherichia coli* [63]. Future studies would need to include this bacteria as well.

Strengths of this study include the large number of samples included, the inclusion of precursor lesions and non-malignant tissue in addition to tumor and paired normal tissue as well as a follow-up investigation investigating the clinical relevance of the bacteria in addition to the bacterial status.

## Conclusion

Our results do not support a role of *S. gallolyticus* in CRC in the Danish population. For *F. nucleatum* and *B. fragilis*, this study does not support a role in development of adenomas, although the bacteria may play a role in the adenoma-carcinoma transition. A potential role of the genera *Prevotella* and *Acinetobacter* in colorectal carcinogenesis was indicated, but warrants further studies.

## Additional files

Additional file 1: qPCR results. Quantification cycles obtained using qPCR and clinical information for each clinical sample, investigated in this study. (XLSX 103 kb)

Additional file 2: Tissue samples used for 16S rRNA gene sequencing. Quantification cycles obtained using qPCR and clinical information for each clinical sample investigated using Illumina sequencing of the V4 region of the 16S rRNA gene. (XLSX 31 kb)

Additional file 3: 16S rRNA gene sequencing metadata. Information describing the samples included for bioinformatics analysis of the 16S rRNA gene sequencing data. (XLSX 11 kb)

Additional file 4: 16S rRNA gene sequences. FASTA file containing the DNA sequences generated using 16S rRNA gene sequencing. (FA 243 kb)

Additional file 5: 16S rRNA gene sequencing OTUtable. OTU table documenting numbers of different OTUs generated during the 16S rRNA gene sequencing. (TXT 163 kb)

## Abbreviations

*B. fragilis*: *Bacteroides fragilis*
CRC: Colorectal cancer
DNA: Deoxyribonucleic acid
*F. nucleatum*: *Fusobacterium nucleatum*
FFPE: Formalin-fixed, paraffin-embedded
HE stain: Hematoxylin and eosin stain
OTU: Operational Taxonomic Units
PCA: Principal component analysis
PCR: Polymerase chain reaction
qPCR: Quantitative real-time PCR
RDA: Redundancy analysis
RNA: Ribonucleic acid
rRNA: Ribosomal RNA
*S. gallolyticus*: *Streptococcus gallolyticus* subspecies *gallolyticus*
